# Establishment of Clonal MIN-O Transplant Lines for Molecular Imaging via Lentiviral Transduction & *In Vitro* Culture

**DOI:** 10.1371/journal.pone.0039350

**Published:** 2012-06-20

**Authors:** David L. Boucher, Jane Qian Chen, Simon R. Cherry, Alexander D. Borowsky

**Affiliations:** 1 Department of Biomedical Engineering, University of California Davis, Davis, California, United States of America; 2 Center for Comparative Medicine, University of California Davis, Davis, California, United States of America; Institute of Cancerology Gustave Roussy, France

## Abstract

As the field of molecular imaging evolves and increasingly is asked to fill the discovery and validation space between basic science and clinical applications, careful consideration should be given to the models in which studies are conducted. The MIN-O mouse model series is an established *in vivo* model of human mammary precancer ductal carcinoma *in situ* with progression to invasive carcinoma. This series of transplant lines is propagated *in vivo* and experiments utilizing this model can be completed in non-engineered immune intact FVB/n wild type mice thereby modeling the tumor microenvironment with biological relevance superior to traditional tumor cell xenografts. Unfortunately, the same qualities that make this and many other transplant lines more biologically relevant than standard cell lines for molecular imaging studies present a significant obstacle as somatic genetic re-engineering modifications common to many imaging applications can be technically challenging. Here, we describe a protocol for the efficient lentiviral transduction of cell slurries derived from precancerous MIN-O lesions, *in vitro* culture of “MIN-O-spheres” derived from single cell clones, and the subsequent transplantation of these spheres to produce transduced sublines suitable for optical imaging applications. These lines retain the physiologic and pathologic properties, including multilineage differentiation, and complex microanatomic interaction with the host stroma characteristic of the MIN-O model. We also present the *in vivo* imaging and immunohistochemical analysis of serial transplantation of one such subline and detail the progressive multifocal loss of the transgene in successive generations.

## Introduction

Ductal Carcinoma *in situ* (DCIS) is the term given to those cancers which demonstrate marked atypia, but are non-invasive. While DCIS without associated invasive breast cancer (IBC) is rarely detected as a palpable lump, it is estimated to account for approximately 20–30% of those cancers found in mammography screening programs [1], [2]. It can be expected that as mammography screening in developing countries becomes more common, so too will the diagnosis of DCIS. While DCIS itself is not generally life-threatening, it is associated with an increased risk of IBC. Conventional wisdom regarding the progression of breast cancer from atypical ductal hyperplasia to DCIS to IBC has recently come into question [3]. However, studies examining the relationship between DCIS, IBC and the molecular pathways that link them, while most definitely needed to enhance treatment of patients showing DCIS, remain underrepresented in the literature.

In order to address this deficiency, we have developed and extensively characterized a mouse mammary intraepithelial neoplasia (MIN) that accurately models human DCIS and progresses to invasive carcinoma. The mouse MIN and the outgrowths derived from their transplantation (MIN-O) satisfy the classic operational definition of premalignancy [4], [5]. Each of the six MIN-O lines that we developed meets the following transplantation criteria: grows in gland-cleared fat pad (orthotopic); does not grow in the subcutis (ectopic); does not senesce over many generations of transplantation; and consistently transforms to a phenotype characterized by an ability to grow in the subcutis (ectopic). The biological transplantability and reproducibility of MIN-O permit accurate experiment-based *in vivo*/*ex vivo* correlations [6], [7], [8] that are not possible in existing xenograft models or in mouse models based on whole mammary glands. These transplantable precancerous MIN-O lines are clonal and have unique morphological and biological characteristics modeling human tissue heterogeneity. Their biological predictability has allowed detailed biological and molecular analyses of transitions from normal to premalignant to malignant and to metastatic states [6], [8], [9], [10], [11]. MIN outgrowths (MIN-O) are easily expandable and amenable to experimental manipulation and the *in vivo* mammary fat pad provides an ideal microenvironment in which to study the complexities of tumor progression. In addition to transplanting and expanding small pieces of MIN tissue for outgrowth, we have shown that MIN-O can arise in a gland-cleared fat pad from a transplanted multicellular sphere or “MIN-O-sphere” derived in culture from a single cell previously isolated from MIN-O donor tissue [12]. To summarize, the MIN-O model, in contrast to more commonly used xenograft and cell line based models, allows experiments to be carried out in immune intact mice with the transition from DCIS to IBC occurring in a microenvironment that more closely recapitulates normal disease progression.

The factors which make the MIN-O model such an attractive model of DCIS unfortunately present challenges in the area of transgene expression. As such, there has been limited utility of this model within the field of molecular imaging where expression of imaging reporter genes can be exploited to track cells and study regulation of gene expression *in vivo*. *In vivo* imaging studies with the MIN-O model have to this point been limited to 2-deoxy-2-[^18^F]fluoro-D-glucose (^18^FDG) studies that track progression based on glucose uptake [13], [14]. In this manuscript, we present a protocol for the efficient lentiviral transduction and *in vitro* culture of “MIN-O-spheres” and the successful transplantation of these spheres which ultimately results in propagation of transgenic MIN-O sublines that retain the *in vivo* properties of this valuable model. The primary goal of this work was to incorporate imaging reporter genes into the MIN-O sublines, thus expanding the reach of MIN-O based molecular imaging studies into areas which uncover the mechanisms and molecular events associated with the progression of DCIS to IBC; however, in a broader sense it is a method that can be employed to rapidly establish clonal transgenic lines derived from a wide range of primary cells.

## Methods

### Ethics Statement

All animal studies were completed in accordance with National Institutes of Health and Institutional Animal Care and Use Committee (IACUC) guidelines. The protocol was approved by the IACUC Committee at the University of California, Davis, under animal protocol #15927.

### Emerald GFP Expression Plasmid

The lentiviral plasmid backbone used for mammalian delivery in this study is a modified form of pLenti6/TR (Invitrogen). Briefly, the sequence containing the cytomegalovirus (CMV) immediate early promoter, β-Globin Intron II and tetracycline receptor was excised and replaced with a customized multiple cloning sequence to allow for subcloning of various promoter/reporter combinations. The EF1α promoter was PCR amplified and subcloned into the pLenti6 vector using *MluI* & *SalI* restriction sites to create pLenti6/EF1α. Next, emerald green fluorescent protein (EmGFP; 487 nm/509 nm) was PCR amplified from pcDNA6.2/EmGFP-miR (Invitrogen) and subcloned into pLenti6/EF1α using *CpoI* restriction sites to create pLenti6/EF1α/EmGFP.

### Preparation of Concentrated Lentiviral Particles

293T cells were obtained from ATCC and cultured in Dulbecco’s Modified Eagle Medium (DMEM) supplemented with 10% fetal bovine serum and 1% penicillin/streptomycin/L-Glutamine (complete DMEM) and maintained at 37°C in the presence of 5% CO_2_ in air. 293T cells were seeded to 15 cm culture dishes and, at approximately 75% confluency, were co-transfected with pLenti6/EF1α/EmGFP and the pLP1, pLP2 and pVSV-G series of viral packaging plasmids (Invitrogen) in a 1∶1∶1∶1 mass ratio. Transfected cells were cultured 48-hours (with a single medium change at 24-hours post-transfection) and supernatant containing lentiviral particles was harvested and filtered through a 0.45 µM acrodisc (PALL Corporation, 4614) to remove cell debris and residual 293T cells. Lentiviral particles were then concentrated 100X by centrifugation in 100 kDa Amicon Ultra-15 centrifugal filter units (Millipore, UFC910024) and used immediately.

### Excision of Precancer Lesion and Preparation of Single Cell Slurry

Excision of all lesions and preparation of single cell slurries were completed as described previously [12]. Briefly, animals were anesthetized with nembitol (60 mg/kg) and opened ventrally to allow for manual removal of precancer lesions. Fatty tissue was trimmed away and the lesion was washed twice with phosphate buffered saline (PBS) and manually minced. The minced lesion was incubated overnight at room temperature in DMEM/F12 media supplemented with BSA fraction V (2%v/v), 5 µg/mL insulin, 0.5 µg/mL hydrocortisone and 3 mg/mL collagenase with constant rocking. The following day, digested cells were subjected to a series of washes, brief trypsinization (0.25 mg/mL trypsin in the presence of 5 µL DNaseI) and were finally resuspended in mammary epithelial growth medium [MEGM, mammary epithelial basal medium supplemented with B27 supplement (5%v/v), epidermal growth factor (20 ng/mL), basic fibroblast growth factor (20 ng/mL) and Heparin (100 µg/mL)] at a concentration of 3.75×10^5^ cells/mL.

### Lentiviral Transduction

In a 1.5 mL microcentrifuge tube, 100 µL of the cell slurry (3.75×10^4^ cells) were mixed with 150 µL concentrated lentivirus. This mix was supplemented with 6 µg/mL polybrene (Chemcon, TR-1003-G) and either centrifuged (30 m/2500×*g*/37°C) or rocked gently for 30 minutes at RT. Cells were then chilled on ice for at least five minutes.

### Plating and Culture of Cells on MatriGel Layer

During transduction of isolated cells, 200 µL of a 2∶1 mix of Growth Factor Reduced MatriGel (BD Pharmingen) and MEGM was applied to wells of a 24-well plate which was then incubated at 37°C for 30 minutes to allow for polymerization of the gel matrix. Following polymerization, 3.5 mL MEGM was mixed with the cell slurry and this mix was applied to the 24-well plate at 1 mL (1×10^4^ cells) per well. Medium was refreshed weekly throughout the period of *in vitro* culture.

### Plating and Culture of Cells within MatriGel Layer

MatriGel (500 µL) was gently mixed with transduced cells and aliquots were applied to either 24-well plates (200 µL; 1×10^4^ cells) or 96-well thin wall PCR plates (10 µL; ∼500 cells). The plates were then incubated at 37°C for 30 minutes to allow for polymerization of the gel matrix. Following polymerization, each well was topped with MEGM (1 mL and 50 µL for the 24- and 96-well plates, respectively) and returned to the incubator. Medium was refreshed weekly throughout the period of *in vitro* culture.

### Identification and Transplantation of Transduced Spheres

Individual spheres were counted and assayed for EmGFP expression using an IX81 inverted fluorescent microscope (Olympus) and images were acquired using a DP72 12.8 MP digital camera (Olympus). Spheres from 24-well plates were identified and transplanted manually with forceps. Spheres from 96-well plates were selected for transplant based both on transgene expression and the presence of only a single sphere per well. This allowed for transplantation of the entire well via syringe following gentle mixing of the entire contents of the well (MEGM and MatriGel). All transplants were made to pre-cleared 4^th^ mammary fat pads.

### In vivo Imaging

At selected intervals, animals were anesthetized with 2% isoflurane and depilatory cream was applied to the general region surrounding the MIN-O lesion. Depilatory cream was removed and animals were placed into the Maestro 2 (Caliper Life Sciences) fluorescent imaging system. For each animal, a monochrome image was first acquired and then a second fluorescent image “cube” was acquired using a blue excitation filter (430–480 nm) and image acquisitions running from 500 nm to 720 nm at 10 nm intervals. This image cube was spectrally unmixed using the accompanying Maestro 2 software. Images presented here are comprised of the unmixed composite image overlaid on the corresponding monochrome image using ImageJ software [15].

### Immunohistochemistry

Four micron sections were prepared from excised MIN-O lesions and either stained with Mayer’s hematoxylin and eosin (H&E) stain or probed with goat α-GFP (1∶800; Abcam; ab6673) followed by horse α-goat (1∶1000; Vector; BA-9500) to detect EmGFP protein expression.

### Quantitative Real Time PCR

EmGFP negative and positive regions were identified by IHC within excised lesions and isolated via laser capture. Genomic DNA was purified from isolated tissue using the DNAeasy purification kit (Qiagen) and subjected to quantitative real time PCR (qPCR) analysis on an iCycler IQ5 (BioRad) using the following primer pairs:

EmGFP 126–310: 5′-CCATCTTCTTCAAGGACGAC-3′

5′-GGCTGTTGTAGTTGTACTCC-3′

PyVmT 825–973: 5′-GACGAAATCCTTGTGTTGCT-3′

5′-ATGTCCAAATACAGATCCTCCA-3′

GAPDH: 5′-TCAAGATCATCAGCAATGCCT-3′

5′-ATCACGCCACAGTTTCCC-3′

Mean Ct values were obtained and subjected to PFAFFL scoring [16] with GAPDH serving as the reference gene.

## Results

### Transduction and in vitro Culture of Isolated Single Cells

In order to optimize the *in vitro* portion of our protocol, two methods of culture were utilized. In the first method, spheres were cultured on a layer of MatriGel that was allowed to polymerize in 24-well plates prior to addition of the transduced cell slurry. In the second method, transduced cells were added to MatriGel prior to plating which allowed for sphere culture within the MatriGel layer. *In vitro* culture of spheres using the second method was completed in 24-well plates and 96-well thin wall PCR plates with similar results relative to sphere formation and transduction efficiency.

**Figure 1:**
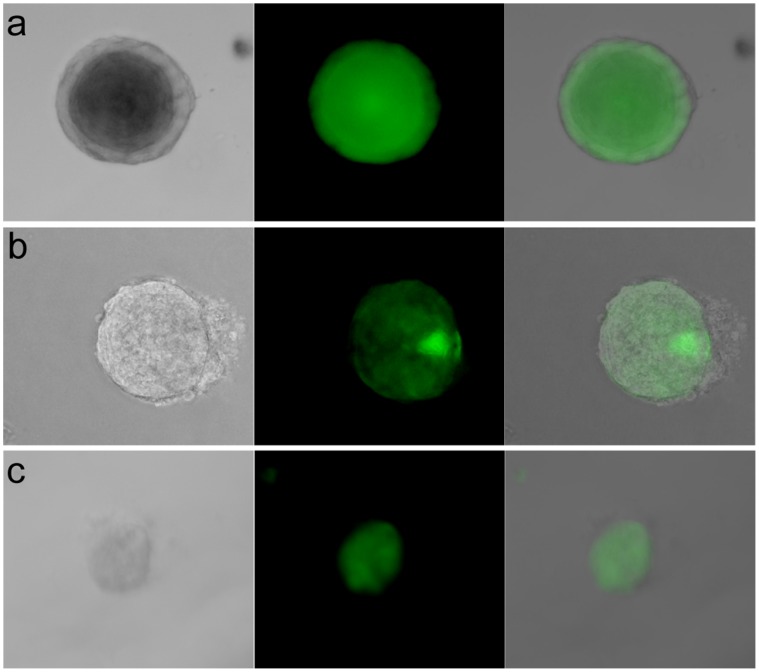
*In vitro* culture of MIN-O-spheres. MIN-O-spheres cultured from single cell slurries of MIN-O Line D grown either on MatriGel in a 24-well plate (a), within the MatriGel layer in a 24-well plate (b) or within the MatriGel layer in a 96-well PCR plate (c). All images are 200X magnification with bright field images shown on the left, corresponding FITC-filtered images in the middle and overlaid images on the right and were captured 14 days post-transduction and plating. While the sphere shown in 1a failed to proliferate following transplantation, both spheres shown in 1b & 1c yielded EmGFP positive lesions *in vivo*.

Approximately four days following plating, early sphere development was evident regardless of culture method. Also at this time, the presence or absence of EmGFP was readily detected in transduced spheres. Although transduction efficiency was rarely less than 40–50%, this figure varied significantly over the course of our studies with a maximum of ∼80% achieved with no significant differences observed between samples subjected to “spinduction” or gentle rocking at RT. Approximately one in 650 isolated single cells formed a sphere *in vitro* when embedded within the MatriGel layer. This is a marked improvement when compared to culture on top of MatriGel which yielded one sphere per approximately 3000 cells. In addition to the increase in sphere formation, sphere morphology was also heavily influenced by culture conditions. Although culture both on top of and within the MatriGel layer yielded multiple sphere types, the relative abundance of various forms was highly dependent upon culture conditions. Following culture on MatriGel, most spheres (>90%) maintained spherical integrity and had what appeared to be a solid core (Figure 1a). However, spheres cultured within MatriGel most frequently displayed a hollow morphology with single sites of irregularity (Figure 1b & 1c). The solid core morphology was never observed in spheres cultured within MatriGel. Additionally, sphere morphology appears to predict growth *in vivo*. The frequency of lesions following transplantation of hollow spheres is typically between 40–80% with variance likely due to the technical challenge of transplantation. Conversely, spheres displaying the solid core morphology have not produced lesions *in vivo* following transplantation (n = 20).

### Transplantation of Single Spheres into FVB Mice

The results presented in the remainder of this manuscript will focus on spheres cultured within the MatriGel layer in 96-well PCR plates. In our experience, this method is the most efficient and least technically challenging of the three methods we have employed. As mentioned previously, *in vitro* culture on MatriGel has not been a successful method of generating spheres suitable for transplantation and *in vivo* propagation. Although we have successfully transplanted spheres cultured within MatriGel in 24-well plates, that method has proven to be highly inefficient due to the technical difficulty of excising only a single sphere and correctly transplanting it into the pre-cleared fat pad.

### First Generation MIN-O-D/EmGFP Transplant Line

**Figure 2:**
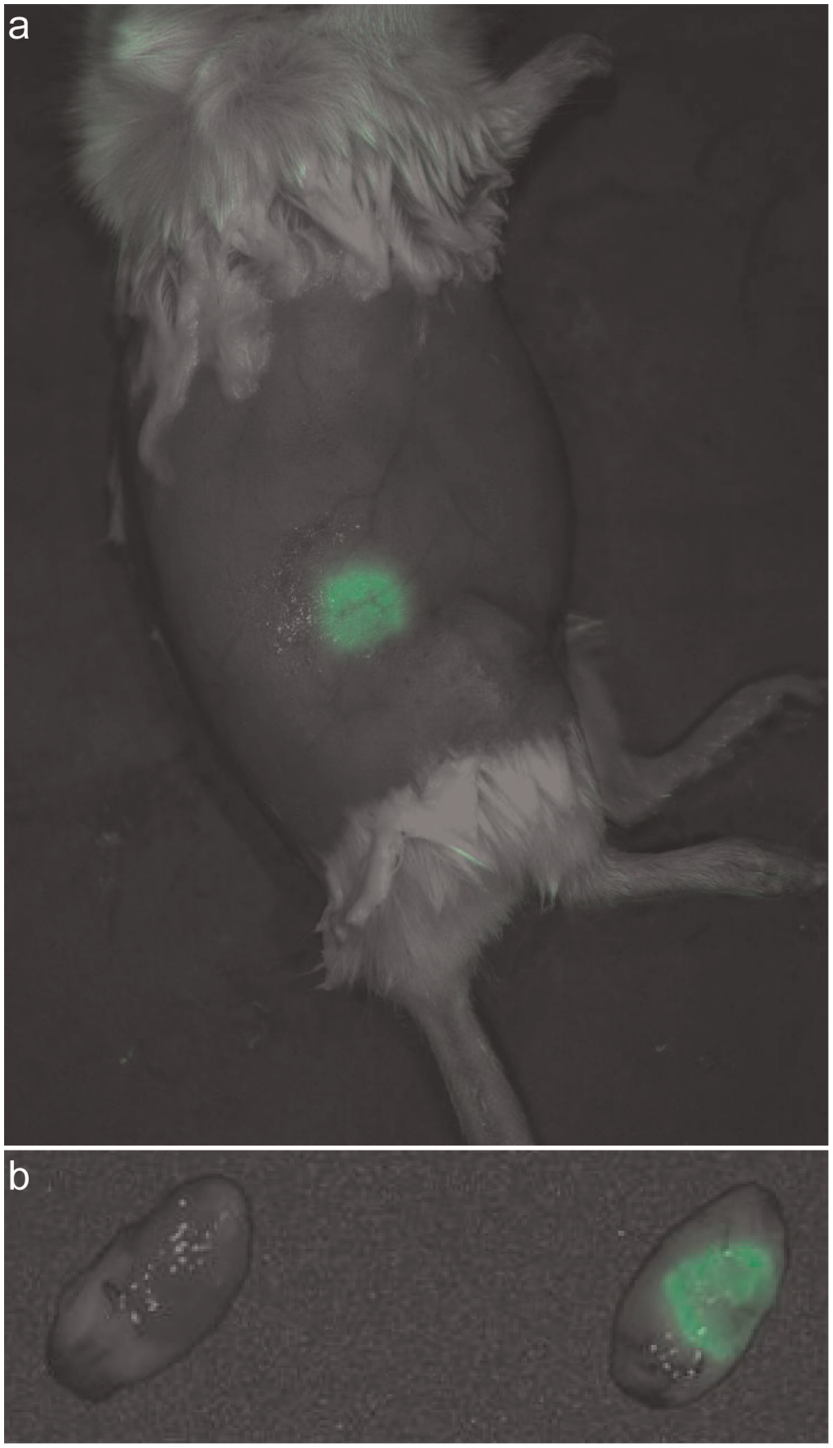
*In vivo* and *ex vivo* images of first generation MIN-O Line D/EmGFP subline. a) Spectrally unmixed image overlaid on the corresponding monochrome image showing the presence of an EmGFP positive lesion in the general region of the 4^th^ right mammary fat pad. b) *Ex vivo* validation of *in vivo* image confirming the source of the EmGFP signal to be the lesion excised from the 4^th^ right mammary fat pad (right). For comparison, an EmGFP-negative lesion is shown on the left. Both images were acquired 27 days post-transplant.

**Figure 3:**
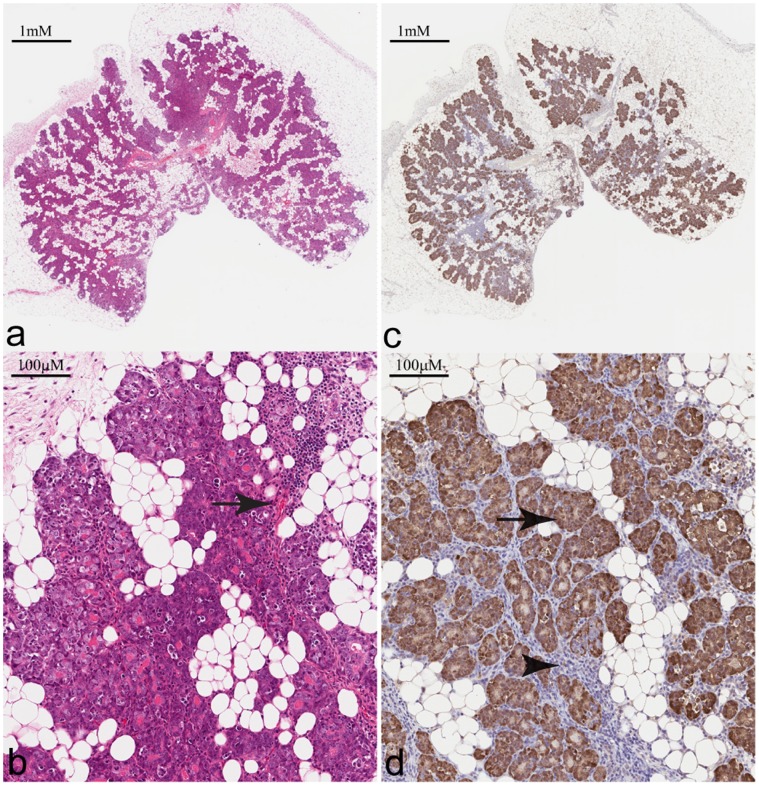
Immunohistochemical analysis of excised MIN-O Line D/EmGFP lesion. Four micron sections of first generation MIN-O/EmGFP lesion either stained with Mayer’s H & E (a&b) or probed with α-GFP in order to asses distribution of EmGFP within the lesion (c&d). Evident in the IHC are morphological characteristics common to the parental MIN-O transplant line which include growth into the fat pad stroma, basal and luminal differentiation, remodeled stroma and increased vascularity (arrow). Analysis of EmGFP expression reveals a high degree of homogeneity throughout the MIN-O tissue (arrow), but not in the host-derived stroma (arrowhead).

Following 14 days of *in vitro* culture, the entire contents of five wells (MatriGel and MEBM; ∼40 µL), each identified to have a single EmGFP positive sphere, were individually transplanted via syringe into pre-cleared 4^th^ mammary fat pads of three FVB mice (bilateral transplants in two mice and a single transplant in the third). By four weeks post-transplant, all five transplants produced lesions; however, the EmGFP signal generated by these lesions was highly variable and in some instances not detectable *in vivo*. Of the five lesions, one in particular displayed a high level of EmGFP signaling *in vivo* (Figures 2a & 2b) and was selected for *in vivo* passage and maintenance. In addition to EmGFP expression, preservation of the characteristics of the MIN-O model was vital. In order to examine this, a portion of the excised lesion was reserved for histologic analysis and immunohistochemistry (IHC). The histology shows MINO morphology characterized by a leading edge of growth into the fat pad stroma, differentiation with basal and luminal epithelial layers in cords and ductules surrounded by remodeled host stroma and increased fat pad vascularity. Progression to central areas of dedifferentiation and proliferation associated with the invasive carcinoma phenotype was also observed. In sum, the morphology and progression was identical to the parent precancer lesion (Figures 3a–d).

### *In vivo* Passage of MIN-O/EmGFP

As the objective of this study is the maintenance of an EmGFP positive subline *in vivo*, a portion of the lesion was disaggregated and a cell slurry was prepared using the remaining portion of the lesion and injected into the pre-cleared fat pad of another FVB mouse. This cycle of excision, dissociation and injection was repeated three times to generate second, third and fourth generation mice. As with the first generation, a portion of each lesion was reserved for IHC analysis.

### Second Generation MIN-O-D/EmGFP

**Figure 4:**
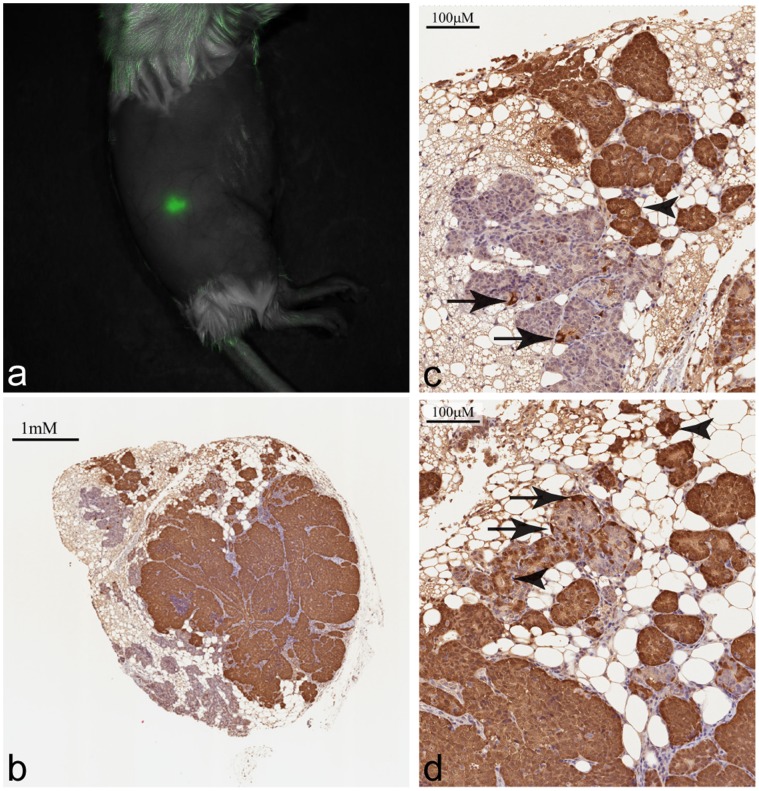
*In vivo* image and immunohistochemical analysis of second generation MIN-O Line D/EmGFP lesion. a) Spectrally unmixed image overlaid on the corresponding monochrome image showing the presence of an EmGFP positive lesion in the general region of the 4^th^ right mammary fat pad. Image was acquired 29 days post-transplant. b) Immunohistochemical analysis of second generation lesion reveals strong general EmGFP expression, but also EmGFP-negative regions. c) Enlarged section of 4b showing a high degree of segregation of EmGFP-positive (upper right) and EmGFP-negative (lower left) cells. d) Enlarged section of 4b showing interspersed EmGFP-positive and EmGFP-negative cells. Arrows show EmGFP positive basal cells and arrowheads show EmGFP positive luminal cells.

At 28 days post-injection of cells isolated from the first generation lesion, the resulting second generation lesion was imaged *in vivo*. As expected, the lesion retained EmGFP expression which was readily detectable (Figure 4a). However, IHC analysis showed that, unlike the first generation lesion, there were small regions of EmGFP negative cells (Figure 4b). These regions included areas in which EmGFP expression was nearly completely lost (Figure 4c) and heterogeneous areas in which EmGFP positive and negative cells were interspersed (Figure 4d).

### Third and Fourth Generation MIN-O-D/EmGFP

**Figure 5:**
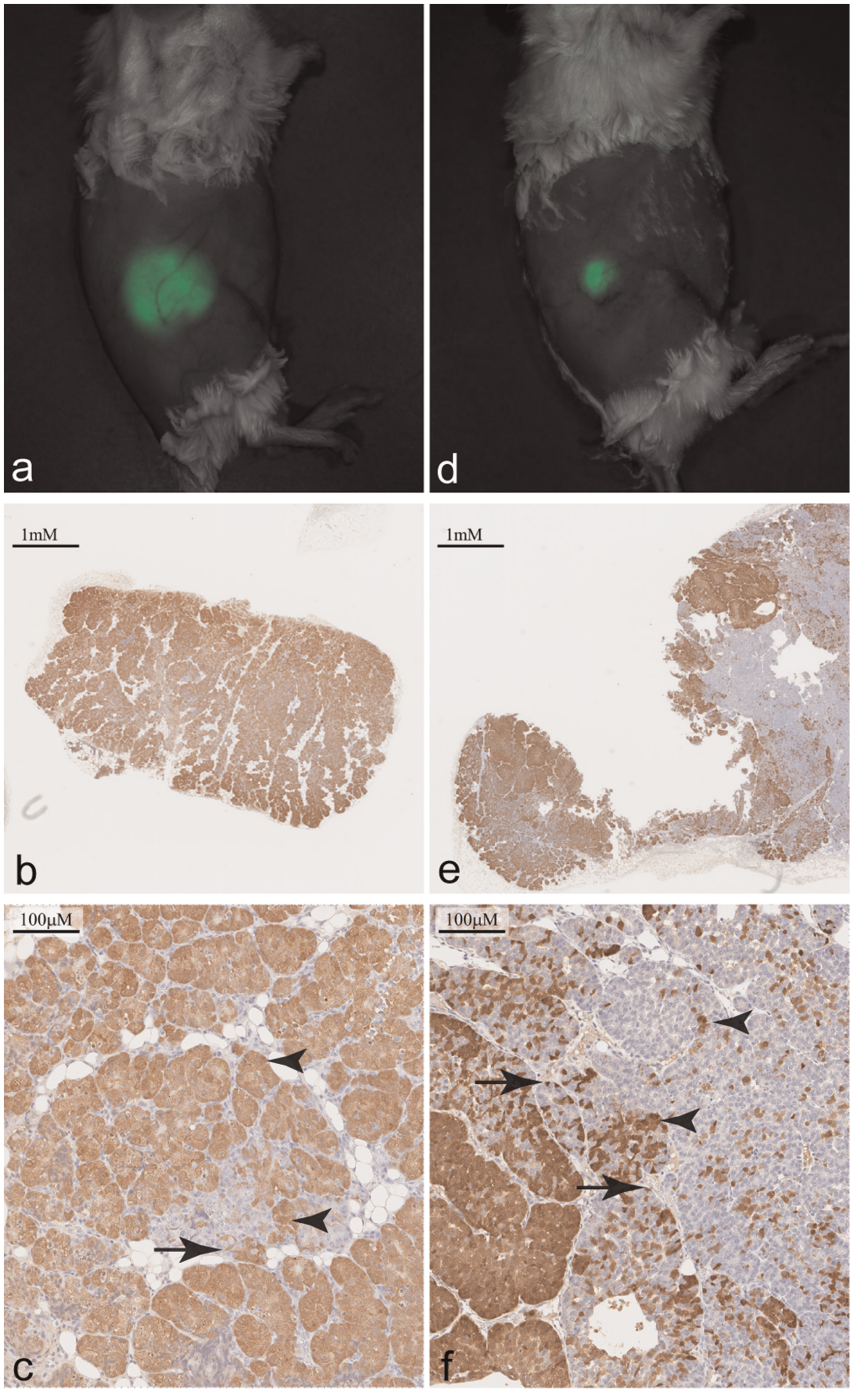
*In vivo* images and immunohistochemical analysis of third and fourth generation MIN-O Line D/EmGFP lesions. a & d) Spectrally unmixed image overlaid on the corresponding monochrome image showing the presence of a GFP positive lesions in the general region of the 4^th^ right mammary fat pad of third (a) and fourth (d) generation MIN-O Line D/EmGFP. Images were acquired 33 and 29 days post-transplant, respectively. b) Immunohistochemical analysis of third generation lesion reveals weakened general GFP expression as well as several GFP-negative regions. e) Similar analysis of fourth generation lesion reveals large regions of GFP-negative cells. c & f) Enlarged sections of 5b and 5f, respectively. Arrows show EmGFP positive basal cells and arrowheads show EmGFP positive luminal cells.

As in both earlier passages, the third and fourth generation MIN-O-D/EmGFP lesions were easily detected with *in vivo* fluorescent imaging (Figures 5a & 5b). Consistent with the IHC results from the second generation lesion, however, a pattern of progressive EmGFP loss was evident in the third and fourth generation lesions (Figures 5c through 5f). Again, this loss presented as regions of complete loss and regions of partial loss. This culminated in massive loss of EmGFP expression in the fourth generation lesion.

### Generational Loss of EmGFP Expression

**Figure 6:**
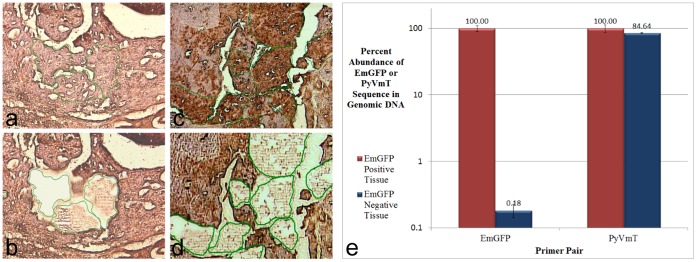
Quantitative Real-Time PCR Analysis of Genomic EmGFP and PyVmT Sequence in Laser Captured EmGFP Positive and Negative Regions. a-d) Images acquired during laser capture of MIN-O tissue shows EmGFP negative regions before (a) and after (b) dissection and EmGFP positive regions before (c) and after (d) dissection. e) qPCR analysis of EmGFP and PyVmT sequence in genomic DNA isolated from laser captured sections. Calculated PFAFFL values were averaged for each primer pair and normalized (EmGFP positive tissue  = 100), allowing for direct comparison of the percent relative abundance of EmGFP and PyVmT sequence in designated samples. While genomic DNA from EmGFP negative tissue shows significant loss of EmGFP coding sequence, a similar decrease in sequence encoding PyVmT was not observed. These data indicate that, while the majority of tissue in both regions is MIN-O in origin, the EmGFP expression cassette has been largely expelled from the genome of EmGFP negative cells.

In order to determine whether loss of EmGFP expression was due to silencing or genomic loss of the lentivirally delivered EmGFP expression cassette, genomic DNA from EmGFP positive and negative regions (isolated via laser capture) was subjected to qPCR analysis using primers for EmGFP and PyVmT. As the MIN-O transplant lines were originally derived from FVB/PyVmT mice, PyVmT is the only known genomic difference between MIN-O-derived tissue and tissue from the otherwise genetically identical FVB host mouse. qPCR analysis of PyVmT in this assay thus confirms whether EmGFP negative tissue is MIN-O-derived or recruited from the host. Results of qPCR showed that, while there is no significant difference in the abundance of PyVmT in EmGFP positive and negative regions, sequence encoding EmGFP is nearly absent (0.18% that of EmGFP positive tissue) in EmGFP negative tissue (Figure 6e). Two conclusions can be drawn from these data. First, tissue from both EmGFP positive and negative areas is predominantly MIN-O in origin (as indicated by the similar prevalence of PyVmT in isolated samples). Second, the dominant mode of observed EmGFP loss is expulsion of the lentivirally delivered expression cassette from the genome rather than gene silencing (as indicated by the absence of EmGFP coding sequence in the genomic DNA of EmGFP negative tissue).

## Discussion

The protocol presented here allows for the development of clonally derived sublines of the MIN-O transplant lines incorporating imaging reporter genes. Prior to this work, non-invasive imaging of this series of lines was limited to studies with administered contrast agents (radioactive or fluorescent) due to an inability to make the type of genomic modifications commonly employed in cell line and xenograft studies. This advance in technology makes possible more sophisticated studies designed to integrate the potential of non-invasive imaging and the biological relevance of MIN-O to elucidate the events involved in progression of DCIS to invasive carcinoma. In addition to the fluorescent reporter construct used here as proof of principle, lentiviral transduction can be readily exploited to deliver a more complex payload such as gene knockdown and overexpression cassettes and/or allele replacement as well as promoter-specific and pathway dependent reporters. The fact that novel sublines are clonal in lineage also allows for greater consistency and clarity of results relative to studies utilizing “mixed pools” of transduced cells.

In addition to the universal advantages inherent in clonal versus mixed pool lines regardless of the cell origin or method of development, we have found that one of the primary advantages of this protocol is the low number of cells required. In the MIN-O model presented here, less than 50,000 cells are required for the plating of a 96-well PCR plate. A single plate yields approximately 12–18 transplantable wells; that is, wells in which only a single fluorescent sphere developed. This was particularly significant within the context of the MIN-O model due to the inability to allow lesions to progress beyond the precancer stage and the resulting decrease in the yield of primary cells per mouse. Given the difficulty and cost associated with culture of many other types of primary cells, we expect this feature of our protocol to be attractive to researchers in need of similarly engineered lines.

Interestingly, sphere morphology is highly predictive of sphere growth *in vivo*. This suggests that there are different types of progenitor cells in the MIN-O, with different biologic potential for differentiation and growth *in vivo*. It is well known that many cell types from the mouse mammary gland show divergent differentiation and growth depending on the culture conditions. In most examples, however, the “on top” versus “in” MatriGel comparisons are equal [17]. In our experiments here, we found that culture within MatriGel favored the transplantable hollow phenotype over the non-transplantable solid core phenotype. While the precise differences between the two culture methods that contribute to differences in sphere morphology are not known, the critical issue within the context of this study is that culture within MatriGel improves the efficiency of developing transplantable MIN-O spheres. Further evaluation of the factors responsible for this increase in efficiency may be important to cancer stem cell differentiation and biology.

Not surprisingly, the primary drawback to serial transplant of genetically engineered cells is the gradual loss of transgene expression with increasing generations. Loss of transgene expression regardless of the mode (i.e., methylation, deletion, loss of heterozygosity, etc.) has been widely accepted as a pitfall of genetically engineered cells. The rate of loss of expression can be highly dependent upon selective pressures (immunogenicity, changes in proliferative rates, etc.) if a given expression cassette and expressed proteins confer negative selection. Even if there is no significant negative selection, transgenes can still be removed or silenced spontanesously. This difficulty is overcome *in vitro* by the maintenance of selective pressure via supplementation of growth media with antibiotics for which resistance is genetically linked to transgene expression. Unfortunately, this approach is not easily achieved within the context of the MIN-O model as the main advantage of this transplant line is that, with the exception of PyVmT and the transgene, MIN-O cells are genetically identical to the host mouse. Fortunately, the utility of the fluorescent reporter during *in vitro* screening can be extended to allow for fluorescence-activated cell sorting (FACS) of cells isolated from precancer lesions in order to enrich for cells in which the transgene remains expressed. In our experience in unrelated projects, we have found that FACS has little or no negative effect on cell viability and lesion formation. Sorting can in theory be completed immediately prior to injection of study cohort(s) to ensure uniform transgene expression as our observations have shown high stability of expression throughout the period of a single generation. Given that the rate of loss of expression will be dependent upon the type of genetic material and the toxicity or immunogenicity of expressed proteins, potential loss of expression should be considered prior to *in vivo* propagation. As a related side note, it is also worth noting that we achieved stable EmGFP expression through a single generation only when the elongation factor 1α (EF1α) promoter was used to drive its expression. We completed several iterations of this protocol using lentiviral particles generated with the pLenti6/CMV/EmGFP plasmid and, although similar results were observed *in vitro* with respect to sphere morphology, transduction efficiency and transgene expression, outgrowths following transplantation were consistently EmGFP-negative indicating “silencing” of the CMV promoter as has been reported extensively [18], [19], [20].

As our understanding of the role microenvironment plays in both normal and atypical development increases, so too will the emphasis placed on the development and utilization of models such as MIN-O which more closely recapitulate the natural processes being queried. As described here, the period from excised unmodified tissue to *in vivo* imaging of genetically modified lesions which are clonal in lineage is approximately 6 weeks. The transduction efficiency of primary cells, notorious for their difficulty to transfect or transduce, is only slightly lower than what is typically achieved in robust cell lines *in vitro*. Because of both of these factors we believe this method of genomic modification of primary cells to be a powerful tool for researchers which will expand the use of complex animal models into areas such as *in vivo* molecular imaging which have been previously largely restricted to studies employing xenografts and cell lines.
